# The BDNF Val66Met polymorphism and health‐related quality of life in youth with obesity

**DOI:** 10.14814/phy2.16140

**Published:** 2024-07-12

**Authors:** Gary S. Goldfield, Jameason D. Cameron, Ronald J. Sigal, Glen P. Kenny, Denis Prud'homme, Mathew Ngu, Angela S. Alberga, Steve Doucette, Diana B. Goldfield, Heather Tulloch, Helen Thai, Kevin R. Simas, Jeremy Walsh

**Affiliations:** ^1^ Healthy Active Living and Obesity Research Group Children's Hospital of Eastern Ontario Research Institute Ottawa Ontario Canada; ^2^ Department of Pediatrics University of Ottawa Ottawa Ontario Canada; ^3^ School of Human Kinetics University of Ottawa Ottawa Ontario Canada; ^4^ Department of Psychology Carleton University Ottawa Ontario Canada; ^5^ Department of Pharmacy Children's Hospital of Eastern Ontario Ottawa Ontario Canada; ^6^ Department of Medicine, Cardiac Sciences and Community Health Sciences, Cumming School of Medicine University of Calgary Calgary Alberta Canada; ^7^ Department of Cellular and Molecular Medicine University of Ottawa Ottawa Ontario Canada; ^8^ University of Moncton Moncton New Brunswick Canada; ^9^ Department of Exercise Science Concordia University Montreal Quebec Canada; ^10^ Department of Community Health and Epidemiology Dalhousie University Halifax Nova Scotia Canada; ^11^ Life Sciences, Queen's University Kingston Ontario Canada; ^12^ Division of Cardiac Prevention and Rehabilitation University of Ottawa Heart Institute Ottawa Ontario Canada; ^13^ Department of Psychology McGill University Montreal Quebec Canada; ^14^ Department of Neuroscience Carleton University Ottawa Ontario Canada; ^15^ Department of Kinesiology McMaster University Hamilton Ontario Canada

**Keywords:** brain derived‐neurotrophic factor (BDNF), health‐related quality of life (HRQoL), obesity, polymorphism, youth

## Abstract

The brain derived‐neurotrophic factor (BDNF) Val66Met polymorphism causes functional changes in BDNF, and is associated with obesity and some psychiatric disorders, but its relationship to health‐related quality of life (HRQoL) remains unknown. This study examined, in youth with obesity, whether carriers of the BDNF Val66met polymorphism Met‐alleles (A/A or G/A) differed from noncarriers (G/G) on HRQoL. The participants were 187 adolescents with obesity. Ninety‐nine youth were carriers of the homozygous Val/Val (G/G) alleles, and 88 were carriers of the Val/Met (G/A) or Met/Met (A/A) alleles. Blood samples were drawn in the morning after an overnight fast for genotyping. HRQoL was measured using the Pediatric‐Quality of Life core version. Compared to carriers of the Val66Met Val (G/G) alleles, carriers of the Met‐Alleles reported significantly higher physical –HRQoL (*p* = 0.02), school‐related HRQoL, (*p* = 0.05), social‐related HRQoL (*p* = 0.05), and total HRQoL (*p* = 0.03), and a trend for Psychosocial‐HRQoL. Research is needed to confirm our findings and determine whether carriers of the BDNF Val66Met homozygous Val (G/G) alleles may be at risk of diminished HRQoL, information that can influence interventions in a high‐risk population of inactive youth with obesity.

## 1 INTRODUCTION

Obesity during adolescence is a serious public health concern given its high prevalence worldwide (World Health Organization, 2021), association with cardiometabolic complications (Freedman et al., 1999), and tracking from childhood to adulthood (DiPietro et al., 1994). Moreover, youth with obesity are often victims of weight‐based teasing and bullying (Goldfield et al., 2010) and other forms of societal stigma, discrimination, and bias (Puhl & Latner, 2007), which has been shown to have untoward consequences on psychological health and well‐being (Eisenberg et al., 2003).

Health‐related quality of life (HRQoL) is an important outcome to study as it embodies the World Health Organization's (WHO) definition of health: to not only include physical health, but also social and emotional well‐being (World Health Organization, 2002). Youth with obesity have lower HRQoL, and longitudinal studies show that obesity is a determinant of lower HRQoL rather than a consequence (Tsiros et al., 2009). Although the etiology of obesity involves a complex combination of genetic, environmental, and social factors (Kopelman, 2000), genetics contribute an estimated 40%–70% of variance in childhood obesity (El‐Sayed Moustafa & Froguel, 2013), with single gene mutations or single nucleotide polymorphisms (SNPs) contributing between 1% and 5% of the variance (Blakemore & Froguel, 2010). Thus, identifying candidate SNPs that predispose youth with obesity to a diminished HRQoL would be important for informing screening and prevention.

Brain‐derived neurotrophic factor (BDNF) is a neurotrophin integral to neuronal development, survival, and plasticity through the tropomyosin‐related kinase B (TrkB) and p75 neurotrophin receptors (Noble et al., 2011). BDNF is expressed in the central nervous system (CNS), primarily in the hippocampus, striatum, brain stem, amygdala, cerebral cortex, cerebellum, and hypothalamus (Baj et al., 2016). Alterations in the valine (val) to methionine (met) substitution in the 5′ promoter‐region of the human BDNF protein, known as the BDNF gene (val66Met single nucleotide polymorphism [G196A; SNP rs 6265]) has a functional impact on BDNF levels via alterations in intracellular processing, trafficking and activity‐dependent secretion that lead to deficiencies of BDNF (Chen et al., 2004; Egan et al., 2003). BDNF impacts many neurotransmitters related to mental disorders (Gratacòs et al., 2007; Hyman et al., 1991), thus, it is not surprising that the BDNF Val66Met polymorphism has been recognized as a potential biomarker for many psychiatric disorders (Gratacòs et al., 2007). Moreover, alterations in BDNF levels as a function of the Val66met polymorphism have also been implicated in appetite regulation and food intake (Lebrun et al., 2006), eating disorders (Chen et al., 2004), obesity (Zhao et al., 2009), and metabolic disorders (Krabbe et al., 2007), providing evidence that BDNF might play an important role in the pathophysiology of these conditions. Many studies have suggested the BDNF Val66Met homozygous Met/Met allele (A/A) or heterozygous Val/Met alleles (G/A) were associated with a reduced risks of certain psychiatric disorders such as substance abuse and anxiety‐related personality traits (Gratacòs et al., 2007; Lang et al., 2005; Sen et al., 2003), although other studies with other conditions show different relationships (Gratacòs et al., 2007). These varying associations may not be surprising given that, although correlated, positive indicators of well‐being and indicators of psychopathology or ill‐being each have unique antecedents and consequences as they represent distinct constructs, and should not be conceptualized as simply falling on opposite ends of the same continuum (Huppert & Whittington, 2003; Ryff et al., 2006). Thus, it is important to empirically examine how these BDNF alleles may be associated with important indicators of wellbeing, such as HRQOL, and not simply extrapolate findings from the psychiatric literature.

To our knowledge, no study has examined the relationship between the Val66Met polymorphism and HRQoL, either in adults or youth, with or without obesity. Accordingly, the aim of this study was to examine, in a sample of inactive youth with obesity, whether carriers of either one or two copies of the Met‐alleles (A/A or G/A) would differ from noncarriers (i.e., Val G/G alleles) on HRQoL dimensions, including Physical‐HRQOL, Social‐HRQOL, Emotional‐HRQOL, School‐HRQOL, and overall HRQOL.

## 2 MATERIALS AND METHODS

### 2.1 Participants

The current study uses a cross‐sectional design, representing a secondary analysis of baseline data from the Healthy Eating Aerobic and Resistance Training in Youth (HEARTY) exercise intervention, which examined the effects of exercise training on percent body fat as the primary outcome, as well as broad set of physiological and psychological health indicators in youth with obesity (Alberga et al., 2012).

Inclusion criteria for participants in the HEARTY study included: being physically active less than 2 days/week, being postpubertal (Tanner stages IV‐V), aged 14–18 years with a BMI >95th percentile for age/sex and/or ≥85th percentile for age/sex with an additional diabetes or cardiovascular disease risk factor as described elsewhere (Alberga et al., 2012). Exclusion criteria included participation in regular or structured exercise or sport activities done more than twice a week for more than 20 min during the previous 4 months, diabetes mellitus, use of any performance enhancing medication, significant weight change (increase or decrease of ≥5% body weight) during the 2 months before enrollment, pregnancy at the start of the study, activity restrictions due to disease (unstable cardiac or pulmonary disease or significant arthritis), other illness (e.g., eating disorders/clinical depression) judged by the participant or study physician to make participation in this study inadvisable.

This study received approval from the Research Ethics Boards at the Children's Hospital of Eastern Ontario (protocol #05/04E) and the Ottawa Hospital Research Institute (protocol #2004219‐01H). Study protocols conformed to the Declaration of Helsinki (World Medical Association, 2013). The study began in March 2005 and was completed in June 2011. Informed consent was obtained from all the individual participants parents or legal guardian or next of kin in order to participate in the study.

The study was registered at: ClinicalTrials.Gov NCT00195858 http://clinicaltrials.gov/show/NCT00195858, September 12, 2005 (Funded by the Canadian Institutes of Health Research).

### 2.2 Design and procedures

For the baseline assessments, the research coordinator performed a complete medical, drug, and physical activity history as well as a physical examination. Clinical interviews were also performed to assess history of dieting, eating disorders, pubertal growth and development, and sedentary activities. Potential participants reporting any history of eating disorder (e.g., uncontrolled binging, binging and purging, anorexia) or clinical depression were excluded from the study (Alberga et al., 2012). Sociodemographic characteristics, pubertal status, lifestyle behaviors, and HRQoL were completed by self‐reported measures in the laboratory, while body composition was quantified using MRI (Alberga et al., 2012).

### 2.3 Measurements

#### 2.3.1 Primary independent variable

BDNFVal66Met polymorphism. BDNFVal66Met Polymorphism‐Val (G/G), Met (A/A), and Val/Met (G/A) alleles. DNA was extracted from twelve‐hour (overnight‐fasting) blood samples of approximately 20 mL of venous blood taken in the morning, from a forearm or antecubital vein and stored in a freezer at −80C. Samples were obtained at baseline before the run‐in period. Isolation of genomic DNA from buffy coat samples was completed following manufactures instructions (FlexiGene DNA Kit (250), Qiagen, Cat No. ID: 51206, Germany). DNA concentration was measured using a spectrophotometer (NanoDrop™ 2000, Thermo Scientific, Waltham, USA). PCR reactions were carried using the following primers (P1: CCTACAGTTCCACCAGGTGAGAAGAGTG, P2: TCATGGACATGTTTGCAGCATCTAGGTA, P3: CTGGTCCTCATCCAACAGCTCTTCTATAAC and P4: ATCATTGGCTGACACTTTCGAACCCA) and the genotyping based on the methods described by Sheikh and colleagues (2010) (Sheikh et al., 2010). The four primers amplify two allele‐specific amplicons (253 and 201 bp) and the entire region as an internal control. The PCR reaction was carried out in a 25 μL reaction volume including, 25 ng of genomic DNA template, the primers, 100 mmol/L of dNTP (Invitrogen, California), 3 mmol/L of MgSO4 (Invitrogen, California), 1× PCRx Amplification Buffer (Invitrogen, California), 1× PCRx Enhancer Solution (Invitrogen, California), and 1 U Taq DNA Polymerase (Invitrogen, California). The PCR cycling conditions used an initial denaturation temperature of 94°C for 5 min, followed by 30 cycles of 94°C for 45 s, 62.5°C for 60 s and 72°C for 60 s, and a final extension step of 5 min at 72°C. PCR amplicons were resolved on a 1.5% polyacrylamide gel, stained with BlueJuice™ Gel Loading Buffer (Invitrogen, California) and visualized on the ChemiDoc™ Gel Imaging System (Bio‐Rad Laboratories, Mississauga, Canada).

#### 2.3.2 Primary dependent variable

Health‐related quality of life. The Adolescent Core version of the Pediatric Quality of Life (PEDSQL) was used to measure HRQoL. This is a 23‐item self‐report survey consisting of generic core scales encompassing physical functioning (eight‐items), emotional functioning (five‐items), social functioning (five‐items), and school functioning (five‐items). A total HRQoL scale score is derived from the mean of all 23 items. A psychosocial HRQoL summary score is derived from the mean of aggregated items from the emotional, social and school functioning subscales. Scores on all scales range from 0 to 100, with higher scores reflecting more positive HRQoL. The PEDSQL has good reliability and it has been validated in healthy youth and with a variety of pediatric clinical populations (Varni et al., 2002), including adolescents with obesity (Schwimmer et al., 2003).

### 2.4 Control variables

#### 2.4.1 Demographic and developmental variables

Background socio‐demographic information was obtained from all participants, including age, sex, ethnicity, and highest level of parental education as measured by self‐report.

#### 2.4.2 Physical activity

Self‐reported physical activity duration was calculated based on the question, “On average, how long do you participate in some sort of physical activity (PA) each day?” with physical activity being cumulative not consecutive. Six response options were provided 1 = <5 min, 2 = 5–15 min, 3 = 15–30 min, 4 = 30–45 min, 5 = 45–60 min, and 6 = >60 min.

#### 2.4.3 Anthropometrics

Height and weight were recorded with a manual stadiometer and scale, respectively, with participants wearing light clothing and no shoes. Waist circumference was measured at a level midway between the lowest rib and the top of the iliac crest, as previously described (Alberga et al., 2012). Body composition was assessed by MRI with a 1.5‐T system (EchoSpeed, signal 11 version; GE Medical Systems). Participants lay prone for whole‐body cross sectional images using protocols by Ross and colleagues (Ross et al., 1992). The MRIs were analyzed using a Slice‐OMaticTM software, version 4.3 (Tomovision, Magog, Canada). Fat‐free mass (FFM) is defined as total lean tissue mass, which includes all fat‐free skeletal muscle, organs, intestines, and bones, without adipose tissue, while fat mass (FM) represents the amount of visceral and subcutaneous adipose tissue. Percent body fat was calculated by dividing the amount of FM by total body mass (FM + FFM) × 100.

### 2.5 Statistical analyses

Baseline characteristics of the sample were computed and are presented in Table 1 using means and standard deviations for continuous data and frequencies and percentages for categorical data. Since the frequency of the homozygous Val66Met Met/Met (A/A) genotype is low (1%–8%) in populations primarily comprised of youth with obesity from European descent (Shen et al., 2018), we combined this group (*n* = 6) with the carriers of the Val/Met (G/A) alleles. These carriers of the Met‐alleles (*n* = 88) were compared to carriers of the homozygous Val/Val (G/G, *n* = 99) genotype on HRQoL dimensions and other continuous variables using independent *t*‐tests or Chi‐Square tests for categorical data. Because there were no group differences on demographic, anthropometric, or behavioral variables, group differences on HRQoL indicators were evaluated by univariate statistics (independent *t*‐tests) to conserve statistical power rather than statistically controlling for these variables using multivariate modeling, which would reduce power unnecessarily. Effect sizes were based on Cohen's d formula, where *d* = 0.20, *d* = 0.50, and *d* = 0.80 indicate small, moderate, and large effects, respectively. The genotype distribution of the current sample was within Hardy–Weinberg equilibrium based on a Chi‐square test (*p* = 0.65) in comparison to a population of youth from European descent (Skledar et al., 2012). Statistical significance was defined as a two‐tailed alpha <0.05. Analyses were conducted using SPSS, version 24.

**TABLE 1:** Characteristics of the Sample by BDNF Val66Met Allele.

	BDNF Val66Met allele		
Variable	Val/Val (G/G) (*n* = 99) allele	Val/met (G/A) (*n* = 82) or met/met (A/A) (*n* = 6) alleles	*p*‐Value
	Mean (SD)	Mean (SD)	
Age (years)	15.5 (1.5)	15.8 (1.3)	0.16
Body Mass Index (kg/m^2^)	34.9 (4.6)	35.2 (4.6)	0.63
Percent Body Fat (MRI^a^)	50 (5.8)	49 (5.9)	0.25
	***n* (%)**	***n* (%)**	
Gender			
Male	29 (29.3%)	33 (37.5%)	0.28
Female	70 (70.7%)	55 (62.5%)	
Ethnicity			
White	71 (71.7%)	68 (77.3%)	0.39
Nonwhite	28 (28.3%)	20 (22.7%)	
Parent Education (Highest)			
High School	19 (19.2%)	13 (14.8%)	0.44
College/University	80 (80.8%)	75 (85.2%)	
Physical Activity (minutes/day)			
<15 min	31 (31.6%)	31 (35.2%)	0.94
15–30 min	26 (26.5%)	23 (26.1%)	
30–60 min	24 (24.5%)	15 (17.1%)	
>60 min	17 (17.4%)	19 (21.6%)	

^a^ MRI, Magnetic Resonance Imaging.

## 3 RESULTS

A total of 187 participants (out of 304 or 62% of the full baseline HEARTY sample) provided informed consent for genetic analysis, had been genotyped for the Valmet66 BDNF, and provided complete HRQoL and demographic data (participants with missing data were excluded from the analyses). Most of the sample (74%) was white. Eighty‐three percent reported coming from parents who completed some university or community college.

Table 1 shows the Valmet66 polymorphism allele frequency breakdown for the sample. Very few participants were carriers of the homozygous (A/A) Met allele (*N* = 6), with approximately equal proportions of participants carrying either the homozygous G/G (*N* = 99) or heterozygous Val/Met (G/A) variants (*N* = 88). There were no significant group differences on age, sex, parental education, ethnicity, adiposity or physical activity. The sample was, on average, 15.5 years old, living with obesity, primarily white and coming from well‐educated parents/families. Approximately 67% of the sample was female. On average, the sample was not physically active.

Table 2 shows that compared to carriers of the Val66Met G/G Allele, carriers of the Met‐Alleles (A/G or A/A/) reported significantly higher physical –HRQoL *t* (181) = 2.29, *p* = 0.02, school‐related HRQoL *t* (180) = 1.96, *p* = 0.05, social‐related HRQoL *t* (180) = 1.96, *p* = 0.05, and total HRQoL *t* (181) = 2.18, *p* = 0.03, and nonsignificant trend on the Psychosocial Summary scale *t* (181) = 1.8, *p* = 0.07. No significant group differences emerged on the emotional HRQoL indicator *t* (181) = 0.69, *p* = 0.49.

**TABLE 2:** Comparison of Health‐Related Quality of Life (HRQoL) Indicators by BDNF Val66Met Allele.

	BDNF Val66Met allele			
Variable	Val/Val (G/G) (*n* = 99) allele	Val/met (G/A) (*n* = 82) or met/met (A/A) (*n* = 6) alleles	*p*‐Value	Effect size
	Mean (SD)	Mean (SD)		
HRQoL^a^				
Physical	69.9 (13.1)	74.3 (13.2)	0.02	0.34
Emotional	65.6 (20.9)	67.7 (19.9)	0.49	0.10
Social	73 (19)	78 (16.1)	0.05	0.26
School	62.3 (18.2)	67.6 (18.3)	0.05	0.29
Psychosocial Summary	67 (15.5)	71.1 (15.3)	0.07	0.26
Total HRQoL	68 (13)	72.2 (13.3)	0.03	0.32

^a^ HRQoL, health‐related quality of life.

## 4 DISCUSSION

The present study was the first to examine the effect of the BDNF polymorphism on HRQoL amongst youth with obesity. We found that compared to carriers of the homozygous Val alleles (G/G), carriers of the Met‐alleles reported significantly greater overall HRQoL, along with several domains of HRQoL, such as Physical, School and Social HRQoL, with a trend toward better Psychosocial HRQoL.

BDNF Met‐allele carriers in our study reported significantly higher physical‐HRQoL than homozygous Val/Val‐carriers. This may not be surprising given that BDNF has been shown to play an important role in locomotor activity in animals (Kernie et al., 2000) and in humans (Marosi & Mattson, 2014). Acute and sustained physical activity increases serum BDNF levels in adults (Dinoff et al., 2016, 2017) as well as in youth with (Lee et al., 2014) and without (Jeon & Ha, 2017) obesity. Interestingly, the more positive perceptions of HRQoL physical functioning and abilities associated with the Met alleles were observed in our study even though physical activity levels did not differ by BDNF genotype. Although no direct comparisons exist in the literature, somewhat consistent findings demonstrated that those with at least one copy of the Met allele reported greater increases in intrinsic motivation during exercise (Caldwell Hooper et al., 2014). Our findings coupled with Caldwell Hooper et al. (2014) suggest that the BDNF gene may influence perceptions about physical competencies and/or the motivational aspects of physical activity. This is an important area of future inquiry, given physical‐self perceptions and abilities predict physical activity duration and intensity (Bauman et al., 2012), raising the possibility that youth with obesity with one copy of the Met alleles could be more adherent to a physical activity intervention.

Given the established role that BDNF plays in brain health and plasticity (Noble et al., 2011), and that the BDNF gene regulates the secretion of BDNF in an activity‐dependent manner (Chen et al., 2004; Egan et al., 2003), the BDNF gene has been implicated in playing an important role in the development of cognition and memory (Egan et al., 2003). Although no studies, to our knowledge, have examined the BDNF gene in relation to academic or school functioning, some studies exist in neurocognition. A few studies showed that carriers of at least one copy of the Met allele performed more poorly on cognitive tasks (Hariri et al., 2003; Savitz et al., 2006). Our finding that Met‐allele carriers reported greater school‐related HRQoL is consistent with those of Foltynie (Foltynie et al., 2005) who reported carriers of the Met allele showed better performance on a measure of executive function, while another study showed null associations (Tsai et al., 2008). Future research is needed to verify if carriers of the Met‐alleles have better or worse academic performance and cognition based on objective measures, as well as perceived attitudes and abilities relating to scholastic behaviors and functioning. We found that Met‐allele carriers reported a greater overall HRQoL, social‐HRQoL and a trend for improved psychosocial HRQoL compared to carriers of the homozygous Val allele. The overall HRQoL scores in both groups were significantly lower than for nonclinical populations of youth, which was expected given the findings that youth with obesity have HRQoL scores that are comparable to youth undergoing cancer treatment (Schwimmer et al., 2003). However, it is important to note that homozygous Val (G/G) carriers scored 4–5 points lower on overall HRQoL and on several sub‐domains, which is a difference considered to be clinically meaningful (Varni et al., 2002). Thus, carriers of the homozygous Val (G/G) allele may be particularly vulnerable to a reduced HRQoL, which is a serious clinical concern given youth with obesity already face widespread weight‐based teasing, bullying and discrimination (Puhl & Latner, 2007), known to lead to greater psychosocial distress and diminished emotional well‐being (Eisenberg et al., 2003; Szwimer et al., 2020). Our findings are consistent with systematic review data showing that carriers of the Met‐allele show a reduced risk of certain psychiatric disorders such substance‐related disorders (Gratacòs et al., 2007) and anxiety‐related personality traits (Lang et al., 2005; Sen et al., 2003), although other studies show Met‐allele carriers are at greater risk of other disorders such as eating disorders and schizophrenia (Gratacòs et al., 2007). This varied pattern of results highlights the complex involvement that BDNF has in the pathophysiology of mental states/disorders.

On a mechanistic level, BDNF is distributed widely in the CNS, including many regions of the brain that regulate mood and behavior. BDNF also gives trophic support for many neurotransmitters known to impact mental disorders (Croll et al., 1994; Hyman et al., 1991; Knusel et al., 1991; Mamounas et al., 1995), and human (Polyakova et al., 2015) and animal (Angelucci et al., 2005) data implicate BDNF in the response to psychotropic medications. Given the regulatory or activity dependent secretions of the BDNF is severely reduced (about 30%) in Met BDNF carriers (Chen et al., 2004; Egan et al., 2003), and the majority of BDNF protein is released from the regulated secretory pathway in neurons (Egan et al., 2003), it has been theorized that Met carriers are at greater risk of depression and other psychiatric disorders given low BDNF is considered a risk factor (Groves, 2007). Although there is consistent support for this hypothesis in the area of eating disorders and schizophrenia (Gratacòs et al., 2007), consistent with our findings, data from population‐based studies and meta‐analyses show that Met carriers have a lower risk of obsessive and compulsive‐related disorders (Gratacòs et al., 2007), while the evidence for relationships to depression is mixed in both youth (Xia & Yao, 2015) and adults (Groves, 2007; Verhagen et al., 2010). This inconsistency has led to calls to revisit the original hypothesis that the hypofunctioning BDNF Met‐alleles serve as biological risk factors for depression and other forms of psychiatric illness or ill‐being (Groves, 2007). One theory postulated to explain these inconsistent results is that the allele (Met vs Val) or their neurobiological expression that confers risk may change across development, resulting in different trajectories and associations between disorders (Casey et al., 2009). More specifically, Carriers of the met‐allele showed greater brain connectivity between the paralymbic neural network and the neocortical associations areas and amygdala, brain regions that govern the processing of sensory and emotional stimuli. Thus, researchers postulate that the greater connectivity between the cortico‐limbic structures may underlie the increased risks associated with this Met‐allele in some disorders (i.e., schizophrenia), while conferring a protective effect against others (i.e., substance abuse, anxiety) (Thomason et al., 2009). In addition, the impact of any single polymorphism on complex diseases (and perhaps HRQoL) may be moderated by demographic factors (Gratacòs et al., 2007; Verhagen et al., 2010) and/or interact with many other environmental factors (i.e., life stress or childhood trauma) (Hosang et al., 2014; Zhao et al., 2018) resulting in complex gene–environment or epigenetic effects. Regardless of the mechanisms, our study is the first to establish that genetic variations in BDNF are associated with HRQoL, an important finding given that good health is not simply reflected in physical health or in the absence of disease, but one that includes good quality of life (World Health Organization, 2002).

### 4.1 Limitations and strengths

Our sample was limited in size, and primarily comprised of white, physically inactive adolescents with obesity presenting for weight loss who were mostly offspring of well‐educated parents, so the results may not be generalizable to all adolescents, with or without obesity, with higher levels of physical activity. Second, our data are cross‐sectional in nature, thus causality cannot be inferred. Lastly, although there were no differences between carriers and noncarriers of the Val66Met‐alleles on demographic, anthropometric and/or lifestyle factors, it is possible additional environmental factors (stress, trauma, etc.) that were not accounted for in our analysis may have impacted the results. The strengths of our study included its novelty, a high risk sample of youth with obesity who exhibit a greater incidence of BDNF‐related complications such as neurocognitive deficits (Liang et al., 2014), metabolic dysregulation (Krabbe et al., 2007), and reduced HRQoL (Tsiros et al., 2009) compared to peers without obesity. Additionally, the PEDS‐QL is a widely used measure of HRQoL that has been validated in healthy and clinical populations of youth (Schwimmer et al., 2003; Varni et al., 2002), strengthening the internal validity of the findings.

## 5 CONCLUSION

In conclusion, we found that carriers of the Met‐alleles of the BDNF Val66Met polymorphism reported statistically and clinically significantly greater overall HRQoL, Social‐HRQoL, School‐related HRQoL, and a trend for better Psychosocial‐HRQoL compared to carriers of the homozygous Val‐allele. Given that youth with obesity typically exhibit lower HRQoL and longitudinal studies show diminished HRQOL is more likely a consequence of obesity rather than a determinant, future research is needed to verify our initial findings and determine whether carriers of this BDNF genotype are genetically predisposed to developing a diminished HRQoL, as well as elucidate the mechanisms that may underlie these relationships. In the era of personalized medicine, this information could potentially inform early intervention efforts in a high‐risk population to promote better quality of life and mental health trajectories during development and into adulthood.

## AUTHOR CONTRIBUTIONS

G.S.G was responsible for conceptualization, funding acquisition, investigation, methodology, and writing—original draft; J.D.C was responsible for writing—review and editing; R.J.S was responsible for conceptualization, funding acquisition, investigation, methodology, project administration and writing—review and editing; G.P.K was responsible for conceptualization, funding acquisition, investigation, methodology, project administration and writing—review and editing; D.P was responsible for writing—review & editing; M.N was responsible for data curation and writing—review & editing; A.S.A was responsible for data curation, project administration, and writing—review & editing; S.D was responsible for data curation, formal analysis, and writing—review & editing; D.B.G was responsible for writing—review and editing; HT was responsible for writing—review & editing; H.T was responsible for writing—review & editing; K.R.S was responsible for writing—review & editing; and Jeremy Walsh was responsible for writing—review & editing.

## FUNDING INFORMATION

The HEARTY trial was supported by grant MCT‐71979 from the Canadian Institutes of Health Research. Dr. Goldfield was supported by a New Investigator Award from the Canadian Institutes of Health Research for part of this trial and subsequently by an Endowed Research Chair from the Children's Hospital of Eastern Ontario Volunteer Association Board. Dr. Sigal was supported by a Health Senior Scholar award from Alberta Innovates‐Health Solutions and previously supported by a Research Chair from the Ottawa Hospital Research Institute during part of this trial. Dr. Kenny was supported by a University Research Chair from the University of Ottawa. Dr. Alberga was supported by a Doctoral Student Research Award from the Canadian Diabetes Association and currently by a FRQ‐S Chercheur Boursier Junior 1 Award.

## CONFLICT OF INTEREST STATEMENT

The authors have no conflicts of interest to declare.

## INSTITUTIONAL REVIEW BOARD STATEMENT

The study was conducted in accordance with the Declaration of Helsinki and approved by Research Ethics Boards at the Children's Hospital of Eastern Ontario (protocol #05/04E) and the Ottawa Hospital Research Institute (protocol #2004219‐01H) in March 2005. The study was registered at: ClinicalTrials.Gov NCT00195858 http://clinicaltrials.gov/show/NCT00195858, September 12, 2005 (Funded by the Canadian Institutes of Health Research).

## INFORMED CONSENT STATEMENT

Informed consent was obtained from all the individual participants parents or legal guardian or next of kin in order to participate in the study.

## Data Availability

The data are available from the corresponding author by reasonable request.
